# Methanol soluble fraction of fruits of *Annona muricata* possesses significant antidiarrheal activities

**DOI:** 10.1016/j.heliyon.2019.e03112

**Published:** 2019-12-31

**Authors:** Nahida Afroz, Md. Ahsanul Hoq, Sharmin Jahan, Md. Mainul Islam, Firoz Ahmed, A.F.M. Shahid-Ud-Daula, Md. Hasanuzzaman

**Affiliations:** a Department of Pharmacy, Faculty of Science, Noakhali Science and Technology University, Noakhali, 3814, Bangladesh; b Department of Microbiology, Faculty of Science, Noakhali Science and Technology University, Noakhali, 3814, Bangladesh

**Keywords:** Biochemistry, Thrombolytic, *Annona muricata*, Antidiarrheal, Methanol

## Abstract

Medicinal plants are the major sources of traditional treatment of disease in Indian subcontinent due to abundant presence of plants and vast side effects of synthetic drug. The present study was subjected to observe *in vitro* thrombolytic, antibacterial, and *in vivo* antidiarrheal activities of methanol soluble fraction of fruits of *Annona muricata*. In thrombolytic activity assay, various concentrations (2 ─ 10 mg/ml) of methanol soluble fraction was used and dose dependently less potent activity was found. The maximum clot lysis 18.33% (p* < 0.05) was achieved at 10 mg/ml of methanolic fruit extract, whereas standard drug streptokinase showed 55.50% (p*** < 0.001) clot lysis. In antibacterial assay, disc diffusion method was used comprising two gram positive (*S. aureus and Micrococcus luteus*) and two gram negative (*E. coli and P. aeruginosa*) bacteria. None of four (0.25, 0.5, 1, and 5 mg/disc) concentration of fruit extract showed antibacterial potentiality, whereas standard amikacin (3 mg/disc) revealed strong antibacterial activities (=~ 23 ─ 24 mm of MIC). To evaluate antidiarrheal activity, castor oil induced diarrhea was created in Swiss albino mice and different doses (100, 200, and 400 mg/kgbw) of fruit extract was introduced post orally. All of three different doses of fruit extract showed significant (p < 0.05 ─ 0.001) antidiarrheal activities. Notably, the percent inhibition of diarrhea by methanolic extract of fruits of *Annona muricata* was found to be 58.38% at a dose of 400 mg/kgbw. The effect of vehicle saline (10 ml/kgbw) was considered as control and loperamide (5 mg/kgbw) as standard that provided 67.01% inhibition of diarrhea. The results suggest that, the fruits of *Annona muricata* possess potent antidiarrheal properties, providing scientific basis of using the plant parts in the treatment of diarrheal disease.

## 1 Introduction

Human beings have used medicinal plants for the recovery of their various diseases for thousands of years (Hill, 1952). Nowadays, especial attention has drawn on phytomedicines as therapeutics giving diverse range of treatment options against diseases. Because of less economic price, less side effects and efficacy in multidrug resistant occurrences, medicinal plants are preferred more useful than synthetic drugs (Ohba et al., 2015; Uddin et al., 2015; Ye et al., 2015). The use of any plant parts such as seeds, berries, roots, leaves, bark, or flowers for medicinal purposes are referred as phytomedicines (Barrett et al., 1999). Formation of thrombus (clot) inside a blood vessel is the main reason of mortality rate in many of the cardiovascular diseases (Katzung, 2007). In Bangladesh, thrombosis is one of the main causes of morbidity and mortality like other developing countries (Islam and Majumder, 2013). Thrombosis leads to acute coronary disorders such as pulmonary emboli, strokes, heart attacks, venous thromboembolic disorders and even to death (Qin et al., 2015). Though alteplase, anistreplase, streptokinase, urokinase and tissue plasminogen activator are wonderful clot lysis agents but still they have significant paucity, including the need for large doses, limited fibrin specificity, bleeding tendency, allergic reactions, and in some cases the thrombi have been proven to be resistant to intravenous tissue plasminogen activator (t-PA) (Bhaargavi and Sindhuja, 2016). For that reason, recently researches are emphasized on traditional and herbal drugs as effective alternatives and numbers of plants have already been indicated to show very outgoing and influential thrombolytic agents (Jakaria et al., 2017).

Mild to life threatening illness can be caused by a large number of bacteria. Urinary tract, surgical wound infections, respiratory infections, skin infections, and gastrointestinal infections etc are the common bacterial infections. In recent years, antimicrobial resistance has become a global problem because of baffling use of antibiotics (Kunin et al., 1987; Cohen, 1992; Bhalodia and Shukla, 2011). Diarrhea is individualized by increased frequency of bowel movements, wet stool and abdominal pains (Ezekwesili et al., 2004). Diarrhea remains the third leading cause of morbidity and mortality among children under five years of age (Chitme et al., 2004). In spite of taking necessary efforts by various international organizations to control this disease, the occurrence of diarrhea all over the world is still high (WHO, 2009). The use of antibiotics as antidiarrheal drugs have significant shortcomings, including adverse effects and microorganisms tend to proceeds resistance toward them (Kouitcheu et al., 2006). For this reason, medicinal plant origin is an important source of new drugs, has continued to be an important area of antidiarrheal research (Soberon et al., 2007). In this manner, advances in phytochemical investigations are effective in curing certain diseases have renewed interest in herbal medicines. *Annona muricata* is an important medicinal plant belonging to the family of custard apple tree called Annonaceae (Moghadamtousi et al., 2015). *Annona muricata*, locally known as soursop which is native to the warmest tropical areas in South and North America and subtropical parts of the world (Coria-Téllez et al., 2018). Soursop is also native to Mexico, Cuba and Central America. Nowadays, this plant is grown in some areas of Southeast Asia and in some Pacific islands. Soursop is usually cultured in foreign country like USA, Mozambique, Somalia and Uganda. Nevertheless, these are commonly cultivated in kitchen garden for its local expenditure (Moghadamtousi et al., 2015). The plant is slender, small, and cold-intolerant trees that grow up to 4–6 m (13–20 feet) tall. The deciduous species have glossy, dark green leaves, the flowers are long, woody and have dark green, juicy, whitish, aromatic fruits (Gajalakshmi et al., 2012). This plant species is not native in Bangladesh and they are found here as cultivated forms. *Annona muricata* is an ancient medicinal plant with rich source of therapeutic constituents. The plant parts were found to contain tannins, phenolic compounds, flavonoids, alkaloids etc (Bento et al., 2013; Souza et al., 2018). Leaves of *Annona muricata* show anticancer, antidiarrheal activity, fruits show antitumor, antiarthritic, antidiabetic, antidepressive activity, bark shows antidiarrheal activity, seeds show neurotoxic, antileishmanial activity. In a recent study, it has been shown that the hydroalcoholic extracts of *Annona muricata* leaves reduced striatal dopamine and norepinephrine, providing sedative hypnotic effects (Souza et al., 2018). Hydroalcoholic extracts of *Annona muricata* leaves was also found to be possesses antiulcerogenic and antimicrobial activities (Bento et al., 2013, 2018). But there were very few research works on this plant, especially in Bangladesh. So, the main objective is to explore the possibility of developing new drug candidates from *Annona muricata* fruit for the treatment of various diseases. And hence, the present study was to observe the possible *in vitro* thrombolytic, antibacterial, and *in vivo* antidiarrheal activities of the crude methanolic extract of *Annona muricata* fruit.

## 2 Materials and methods

### 2.1 Plant material

The fresh fruits of *Annona muricata* were collected from Sylhet, Bangladesh in the month of February 2016. It was authenticated by National Herbarium Institute, Mirpur, Dhaka, Bangladesh. A voucher specimen of the sample has been preserved as accession number ACC-DACB-45116.

### 2.2 Drugs and chemicals

All chemicals and reagents used in this study were of analytical grade. Methanol (99.5%), which was used for extraction, was procured from Sigma-Aldrich (Hamburg, Germany). Standard streptokinase was purchased from Popular Pharmaceuticals Limited, Bangladesh. During conducting an antibacterial assay of plant extracts amikacin was procured from Square Pharmaceuticals Limited, Bangladesh (Kaliakoir, Gazipur, Bangladesh). The bacterial strains were collected from the American type culture collection (ATCC). Loperamide (Square Pharmaceuticals Ltd., Bangladesh), castor oil (WELL's Health Care, Spain), normal saline solution (0.9% NaCl, Shazib Pharmaceuticals Limited) were also used in this study.

### 2.3 Plant extraction

After collection the fruits of *Annona muricata* were thoroughly washed with water to remove undesirable materials. Then the collected fruits were sun dried at least one week and ground into fine powder with a suitable grinder. About 600 g of powdered materials were soaked in 2300 ml of methanol (80%) and kept at room temperature for 2 weeks. Then it was filtered through whatman filter paper (Bibby RE200, Sterilin Ltd., UK) and evaporated by using rotary evaporator. It rendered a gummy concentrate of brownish black color. The gummy concentrate was designated as crude extract of methanol.

### 2.4 Experimental animals

All animal procedures and experimental protocols were approved by the Research Ethics Committee of Department of Pharmacy, Noakhali Science and Technology University (Ref-2018/BKH1303046F). The experiment was accomplished by using healthy Swiss albino mice of either sex (male and female), weighing 20–30 g and aged 6–8 weeks. All of the animals were procured from the animal house of the Department of Pharmacy, Jahangirnagar University, Savar, Bangladesh. The animals were sheltered in plastic cages at room temperature and on a 12 h light-dark cycle and acclimatized for one week before the inauguration of the experiment. During the whole period of the study, the animals were supplied with standard pellet diet and tap water ad libitum. Prior to the experiment, animals were fasted for 18 h during which food but not water was warded-off. Care and handling of the animals were according to internationally permitted guidelines (NRC, 2011).

### 2.5 Thrombolytic activity

The thrombolytic activity of fruit extract of *Annona muricata* was assessed by the method developed Prasad (Prasad et al., 2006) with little modification. Human blood samples were collected from healthy volunteers who didn't have a history of oral contraceptive or anticoagulant therapy. Seven ml of venous blood was collected from healthy volunteers (n = 3) for this investigation and shifted to different sterilized microcentrifuge tube (1 ml/tube) which were pre-weighted. Then the microcentrifuge tubes were incubated for 45 min at 37 °C. After clot formation, serum was fully disallowed from the tubes and each tube having clot was again weighed to determine the clot weight (clot weight = weight of clot containing tube – weight of tube alone). Each microcentrifuge tube containing clot was entirely labeled. The plant extract was dissolved in methanol and shaking vigorously on a vortex mixer. After vortexing, the different concentrations (2, 4, 6, 8, 10 mg/ml, respectively) of the test samples were prepared. Then the suspension was placed nightly and blended to remove the soluble supernatant and then filtered through a 0.22- micron syringe filter. After filtration, 100 μl of methanolic extract was added to each labeled tube (2─10 mg/ml). At the same time, 100 μl of streptokinase and 100 μl of distilled water were distinctly added to the tubes containing clot that served as positive control and negative thrombolytic control, respectively. All the tubes were then incubated for 90 min at 37° and observed for clot lysis. Finally, difference obtained in weight taken before and after clot lysis was revealed as percentage of clot lysis (Prasad et al., 2006).

$$\text{\%}\;\text{of}\;\text{clot}\;\text{lysis}=\frac{\text{Weight of released clot}}{\text{Clot weight}}\times 100\text{\%}$$

### 2.6 Antibacterial activity

#### 2.6.1 Test microorganisms

For the ascertainment of antibacterial activities of methanolic extract of *Annona muricata* fruits, four variant microorganisms were used. Two of species were Gram-negative bacteria (*Escherichia coli* ATCC 25922 and *Pseudomonas aeruginosa* ATCC 15422) and other two was Gram-positive bacteria (*Micrococcus luteus* ATCC 4698 and *Staphylococcus aureus* ATCC 2592). Antibacterial activities were ascertained by using disk diffusion method as described by Sacchetti (Sacchetti et al., 2005). The disk diffusion method is ascertained using Muller-Hinton Agar (MHA), which is the excellent medium for routine susceptibility, low in sulfonamide, trimethoprim and tetracycline inhibitors, and gives favorable growth of most bacterial pathogens (Alderman and Smith, 2001).

#### 2.6.2 Procedure

Four petridishes were streaming with Muller- Hinton Agar (MHA) media which were formerly sterilized. After induration of the media in culture plate, commercial bacteriological loops containing test microorganisms were used to seed the selected microorganism. Then the plates were incubated at 37 °C for 24 h. The haphazard of each culture was then fixed to a similar optical density to that of McFarland 0.5. The ascertained cultures were inoculated onto agar plates by swabbing equally over the whole surface of the medium. Sterile paper discs (6 mm diameter, made from Whatman No. 1 filter) were saturated with 10 μL of plant extract (10–500 μg/mL) and distilled water and engrossed carefully on the surface of the inoculated agar plate with slight pressure. As a positive control, standard antimicrobial disks (amikacin, 3μg/disc) were used. To allow optimal growth of microorganism, the dishes were then incubated at 37 °C for 24 h. If the test sample is acquainted of any antibacterial activity, it will abate the growth of the microorganisms and a translucent, distinct zone of inhibition will be emerged surrounding the medium. The antibacterial activity of the test sample was enumerated by measuring the diameter of zone of inhibition revealed in millimeter (Ferdous et al., 2018).

### 2.7 *In vivo* antidiarrheal activity

#### 2.7.1 Castor oil-induced diarrhea

Anti-diarrheal activity of fruit extract of *Annona muricata* was investigated by castor oil induced method following Awouters et al. and Mukherjee et al. with little modification (Awouters et al., 1978; Mukherjee et al., 1998). For the experiment, mice were starved for18 h before the test with free accession to water. Mice are divided into control, positive, and test groups containing seven mice in each group. Control group received vehicle (normal saline solution, post orally) at a dose 10 ml/kgbw. The positive control group received loperamide at a dose of 5mg/kgbw. The test group received methanolic extracts at the doses of 100, 200, and 400 mg/kgbw. After 30 min of administration of test samples, the mice of all groups were orally treated with 0.2 ml of castor oil to induce diarrhea. Then the mice were kept individually in cages lined with white blotting paper and observed for 5 h. The following parameters were observed: time to initial evacuation (min), evacuation classification: 1 (normal stool), 2 (semi-solid stool), 3 (watery stool) and evacuation index (EI). Evacuation index was determined according to the following formula:

EI=1×(nstool1)+2×(nstool2)+3×(nstool3)

### 2.8 Statistical analysis

The results were presented as mean ± standard error of mean (SEM). The one-way ANOVA with Dunnett's post hoc test was used to analyze and compare the data using GraphPad Prism version-5 (GraphPad Software, San Diego California USA)., while *p* ≤ 0.05–0.001 were considered statistically significant value.

## 3 Results and discussion

### 3.1 Antibacterial activity

The results of methanolic extract of *Annona muricata* on antibacterial screening have been shown in Table 1. The methanolic extract of *Annona muricata* fruit did not exhibit any effective antibacterial activity against the tested bacteria. As none of the concentrations of methanolic extract of *Annona muricata* fruits showed antibacterial activity against Gram-positive (*S. aureus and Micrococcus luteus*) spectrum and also even in Gram-negative bacterial (*E. coli and P. aeruginosa*) spectrum, it can be said that the test sample has no antibacterial activity against both the gram positive and gram negative bacteria.

**Table 1:** Antibacterial activities of methanolic extract of fruits of *Annona muricata*.

Microorganism	Zone of inhibition (mm)				
	Standard (Amikacin)	Fruits extract			
	3 mg/disc	5 mg/disc	1 mg/disc	0.5 mg/disc	0.25 mg/disc
**Gram Negative**					
*Pseudomonas aeruginosa*	23 ± 00	-	-	-	-
*Escherichia coli*	23 ± 00	-	-	-	-
**Gram Positive**					
*Micrococcus luteus*	19 ± 0.50	-	-	-	-
*Staphylococcus aureus*	24 ± 0.29	-	-	-	-

Values are Mean ± SEM (n = 3).

In this study, extracts of *Annona muricata* fruits were invented to neither active against gram positive bacteria nor gram negative bacterial strains. Similar observation was also indicated in a study where methanolic extract of *Annona senegalensis* did not show measurable activity against *E.coli*. In contrast, *Annona senegalensis* is active against *P. aeruginosa* or *S. aureus* (Lino and Deogracious, 2006). Similarly, methanolic extract of *Annona reticulata* bark showed significant activity against *Staphylococcus aureus* (Padhi et al., 2011). There is no such study of *Annona muricata* regarding antibacterial activities; however one study reported that the plant extract of *Polyalthia longifolia* (Annonaceae) has no or very little antibacterial properties (Valsaraj et al., 1997) which is somehow correlates our findings.

### 3.2 Thrombolytic activity

The effects of *Annona muricata* fruits extract on in-vitro clot lysis are shown in Table 2. It is pronounced that the percentage of clot lysis was 55.50 ± 0.71% when 100 ml of streptokinase (30,000 I.U.) was used as a positive control, while in the case of distilled water (negative control) the percentage of clot lysis was only 3.6 ± 0.83%. The methanolic extracts of *Annona muricata* fruits showed 8.98 ± 4.08, 11.87 ± 7.32, 12.72 ± 3.92, 17.67 ± 6.086 and 18.33 ± 3.87% of clot lysis at a concentration of 2,4,6,8, and 10 mg/ml, respectively. The percentage of clot lysis was dose-dependently increased, whereas 10 mg/ml concentration showed maximum clot lysis that was 18.33 ± 3.87%.

**Table 2:** The Effects of different concentration of methanolic extract of fruits of *Annona muricata* on *in-vitro* clot lysis.

Treatment	% of clot lysis of *Annona muricata*
Blank	3.6 ± 0.83
Streptokinase	55.50 ± 0.71***
ME (2 mg/ml)	8.98 ± 4.08
ME (4 mg/ml)	11.87 ± 7.32
ME (6 mg/ml)	12.72 ± 3.92
ME (8 mg/ml)	17.67 ± 6.086
ME (10 mg/ml)	18.33 ± 3.87*

The statistical analysis was carried out using one way ANOVA. Here, ME stands for methanolic extract and all the values are expressed as mean ± SEM of three independent experiment where, level of significance stated as for P*** <0.001, and P* < 0.05. Distilled water was employed as negative control and streptokinase was employed as positive control. Comparison was observed in between blank and standard as well as blank and test group.

Until now, a large number of research works have been disclosed that plants and natural food sources restrain coronary events and stroke (Das et al., 2013). The current study was an endeavor to find out the clot lysis potentiality of methanolic extract of *Annona muricata* fruits. The comparison of the result of positive control (streptokinase) with that of negative control (distilled water) undoubtedly revealed that clot disruption does not occur when distilled water was added to the clot. The mean difference in clot lysis percentage between positive and negative control was significant (p value <0.001). The observed result indicated that five different test samples of plant has negligible amount of clot disruption compare to positive control.

Very recently, deposition of platelets, tissue factor, and fibrin through thrombosis or blood clot formation block cell surface or blood vessels which become a troublesome event (Furie and Furie, 2008). By breaking down the fibrinogen and fibrin contained in clot, thrombolytic drugs block the pathway of thrombus formation with the help of plasmin that lyse the clot. Clot lysis are increased by commercial thrombolytic drugs such as streptokinase, that convert plasminogen to plasmin (Mbagwu and Adeyemi, 2008). Plasmin is the most common natural anti-thrombotic agents, which is itself aroused from cell surface bound plasminogen which is then ultimately conduct to fibrinolysis (Pantzar et al., 1998). Nowadays large number of research works have been undertaken to discover antithrombotic agents (anticoagulant and antiplatelet) from plants and natural food sources in order to the prevention of coronary events and stroke (Hussain et al., 2016). It was indicated that phytochemicals like saponin, alkaloids, and tannin are accountable for the thrombolytic activity (Rahman and Das, 2013). As the methanolic fruits extract of *Annona muricata* do not possess any saponin, and tannin, it might be the reason of low or mild thrombolytic activity of this plant parts (Rosniza et al.). However, the presence of alkaloids (our unpublished data) might be the responsible for mild thrombolytic activity.

### 3.3 Antidiarrheal activity

Based on ethnopharmacological data, the effect of methanolic extracts of fruits of *Annona muricata* on diarrhea was evaluated. The results for castor-oil induced diarrhea are summarized in Table 3. All of the aliquot of extract of *Annona muricata* fruits were found to be effective in a dose dependent manner against castor oil induced diarrhea on experimental mice. At the dose of 400 mg/kgbw, the extract showed significant antidiarrheal activity (P < 0.001) showing 58.38 ± 9.49% reduction of diarrhea that is comparable to that of standard drug loperamide (67.01 ± 9.96% inhibition of diarrhea).

**Table 3:** Effects of methanolic extract of fruits of *Annona muricata* on castor oil induced diarrhea in mice.

Treatment		Vehicle	Loperamide	ME (*Annona muricata*)		
Dose (mg/kgbw)		10 (ml/kgbw)	5	100	200	400
N		7	7	7	7	7
Time to initial evacuation		29.57 ± 29.57	138.85 ± 24.94	30.00 ± 30.00	30.43 ± 30.43	98.00 ± 47.02
Evacuation classification	Normal	1.86 ± 0.34	2.86 ± 0.55	1.00 ± 0.44	2.14 ± 0.46	2.43 ± 0.37
	Semi-solid	3.00 ± 0.23	0.86 ± 0.40**	0.43 ± 0.43**	1.29 ± 0.64	1.29 ± 0.47
	Liquid	1.86 ± 0.34	0.00 ± 0.00*	2.71 ± 0.29	0.71 ± 0.47	0.29 ± 0.18*
Evacuation Index (EI)		14.29 ± 0.99	4.57 ± 1.27***	10.00 ± 0.44*	6.86 ± 1.81*	5.86 ± 1.34**
Inhibition (%)		-	67.01 ± 9.96	28.09 ± 5.44	53.26 ± 11.45	58.38 ± 9.49

Data are presented as Mean ± SEM; N = 7 in each group. ANOVA was employed, followed by Dunnett's test, and significant difference were presented by p*** < 0.001, p** < 0.01, p* < 0.05 vs control group treated with vehicle.

All mice from the control group (treated with vehicle) produced copious diarrhea after castor oil administration which is demonstrated by higher evacuation index (Table 3). This condition was markedly reduced by loperamide that is shown as 67.01 ± 9.96% diarrheal inhibitions. Oral pre-treatment of mice with methanolic extract (100, 200 and 400 mg/ml) showed a significant delay in the onset of the copious diarrhea (28.09 ± 5.44, 53.26 ± 11.45 and 58.38 ± 9.49%, respectively, relative to the vehicle). The higher dose of the extract (400 mg/kg) exhibited better effect compared to other groups of extract (Table 3).

The aim of the present study was to investigate the antidiarrheal activity of the methanolic fruit extract of *Annona muricata* by treating different dose of extract in castor oil induced mice. In the upper part of the small intestine, ricinoleic acid is released by the action of lipases (Kulkarni and Pandit, 2005). By binding to EP3 prostanoid receptors on smooth muscle cells, it interposes its action (Tunaru et al., 2012) and by suppressing absorption and enhancing secretion of fluid and electrolytes, it naturalizes the accumulation of fluid in the intestine (Racusen and Binder, 1979). Moreover, the motility of GI smooth muscles is altered by the metabolites of castor oil (Mathias et al., 1978). The motto of the inhibition of castor oil induced diarrhea can therefore be connected with the deduction of the synthesis of all or either of those mediators by the experimental extract. This suggestion is affirmed by the facts that stimulation of prostaglandins biosynthesis is related to castor oil induced diarrhea (Robert et al., 1976; Taufiq-Ur-Rahman et al., 2005). A number of researchers have demonstrated the flavonoids, triterpenoids, saponins, especially flavonoid possesses ability to avert intestinal motility and hydroeletrolytic secretions which are known to be altered in diarrheic conditions (Gaginella et al., 1975). Since *Annona muricata* fruit is known to high content of flavonoid ((Rosniza et al.) and our unpublished data), it is reasonable to have antidiarrheal properties on its plant parts.

## 4 Conclusion

These findings indicate that the plant may be a potential source for the development of new antidiarrheal drug. The obtained results revealed that the methanolic fruit extract of *Annona muricata* possessed with significant antidiarrheal activity. It generates an inhibitory effect on castor oil induced diarrhea. The acquired results also ascertained the presence of less potent thrombolytic effect. However, the extract didn't exhibit any antibacterial activity.

## Declarations

### Author contribution statement

Nahida Afroz, Md. Ahsanul Hoq: Performed the experiments; Wrote the paper.

Sharmin Jahan: Analyzed and interpreted the data.

Md. Mainul Islam: Analyzed and interpreted the data; Wrote the paper.

Firoz Ahmed: Contributed reagents, materials, analysis tools or data.

A. F. M. Shahid-Ud-Daula: Conceived and designed the experiments.

Md. Hasanuzzaman: Conceived and designed the experiments; Analyzed and interpreted the data; Wrote the paper.

### Funding statement

This research did not receive any specific grant from funding agencies in the public, commercial, or not-for-profit sectors.

### Competing interest statement

The authors declare no conflict of interest.

### Additional information

No additional information is available for this paper.
